# Empowering rural communities for effective larval source management: A small-scale field evaluation of a community-led larviciding approach to control malaria in south-eastern Tanzania

**DOI:** 10.1016/j.parepi.2024.e00382

**Published:** 2024-10-04

**Authors:** Salum A. Mapua, Alex J. Limwagu, Dmitry Kishkinev, Khamis Kifungo, Ismail H. Nambunga, Samuel Mziray, Gwakisa John, Wahida Mtiro, Kusirye Ukio, Javier Lezaun, Frederic Tripet, Fredros O. Okumu

**Affiliations:** a Environmental Health and Ecological Sciences Department, Ifakara Health Institute, P. O. Box 53, Morogoro, Tanzania; b School of Life Sciences, Keele University, Huxley Building, Keele, Staffordshire ST5 5BG, UK; c Tanzania Biotech Products Limited, P. O. Box 30119, Kibaha, Pwani, Tanzania; d President's Office-Regional Administration and Local Government, Morogoro Regional Secretariat, P.O. Box 650, Morogoro, Tanzania; e Institute for Science, Innovation and Society, School of Anthropology and Museum Ethnography, University of Oxford, 64 Banbury Road, Oxford OX2 6PN, UK; f Swiss Tropical and Public Health Institute, Kreuzgasse 2, 4123 Allschwil, Switzerland; g School of Public Health, Faculty of Health Sciences, University of the Witwatersrand, Johannesburg, South Africa; h School of life science and bioengineering, The Nelson Mandela African Institution of Science and Technology, P. O. Box 447, Arusha, Tanzania; i School of Biodiversity, One Health and Veterinary Medicine, University of Glasgow, Glasgow, G61 1QH, UK

**Keywords:** Malaria control, Larviciding, Larval source management, Biolarvicides, Community engagement, Tanzania, Ifakara health institute

## Abstract

**Introduction:**

Larval source management, particularly larviciding, is mainly implemented in urban settings to control malaria and other mosquito-borne diseases. In Tanzania, the government has recently expanded larviciding to rural settings across the country, but implementation faces multiple challenges, notably inadequate resources and limited know-how by technical staff. This study evaluated the potential of training community members to identify, characterize and target larval habitats of *Anopheles funestus* mosquitoes, the dominant vector of malaria transmission in south-eastern Tanzania.

**Methods:**

A mixed-methods study was used. First, interviewer-administered questionnaires were employed to assess knowledge, awareness, and perceptions of community members towards larviciding (*N* = 300). Secondly community-based volunteers were trained to identify and characterize aquatic habitats of dominant malaria vector species, after which they treated the most productive habitats with a locally-manufactured formulation of the biolarvicide, *Bacillus thuringiensis* var. *israelensis*. Longitudinal surveys of mosquito adults and larvae were used to assess impacts of the community-led larviciding programme in two villages in rural south-eastern Tanzania.

**Results:**

At the beginning of the program, the majority of village residents were unaware of larviciding as a potential malaria prevention method, and about 20 % thought that larvicides could be harmful to the environment and other insects. The trained community volunteers identified and characterized 360 aquatic habitats, of which 45.6 % had *Anopheles funestus*, the dominant malaria vector in the area. The preferred larval habitats for *An. funestus* were deep and had either slow- or fast-moving waters. Application of biolarvicides reduced the abundance of adult *An. funestus* and *Culex* spp. species inside human houses in the same villages, by 46.3 % and 35.4 % respectively. Abundance of late-stage instar larvae of the same taxa was also reduced by 74 % and 42 %, respectively.

**Conclusion:**

This study demonstrates that training community members to identify, characterize, and target larval habitats of the dominant malaria vectors can be effective for larval source management in rural Tanzania. Community-led larviciding reduced the densities of adult and late-stage instar larvae of *An. funestus* and *Culex* spp. inside houses, suggesting that this approach may have potential for malaria control in rural settings. However, efforts are still needed to increase awareness of larviciding in the relevant communities.

## 1 Introduction

Since 2000, malaria incidence and mortality have significantly decreased due primarily to expanded vector control with insecticide-treated nets (ITNs) and indoor residual spraying (IRS), along with improved diagnosis and treatment (Bhatt et al., 2015). However, these gains began plateauing around 2015, and many high-burden countries now report increases in cases (WHO, 2023). The latest estimates from the World Health Organization (WHO) indicate that the disease still causes around 249 million cases and 608,000 deaths, with a majority of this burden being in Africa (WHO, 2023).

In addition to poor socio-economic circumstances and the generally weak health systems in endemic communities (The malERA Consultative Group on Health Systems, 2011; Okumu et al., 2022), the continued burden of malaria is also associated with major biological threats such as the widespread resistance of malaria vectors to insecticides (Hancock et al., 2020; Moyes et al., 2020) and behavioral resistance to indoor interventions (Russell et al., 2011; Moiroux et al., 2012; Sougoufara et al., 2014), as well as growing tolerance of the malaria parasite (*Plasmodium* spp.) to anti-malarial drugs. Human behaviors, such as staying outdoors unprotected during times of increased malaria transmission, also contribute to ongoing malaria transmission (Finda et al., 2019; Matowo et al., 2013; Monroe et al., 2015). Another important but often less discussed challenge is that sustained malaria prevention is untenable without sufficient engagement of local communities and other stakeholders (Adhikari et al., 2020; Asale et al., 2019).

Research has shown that involving rural communities in vector control programs is beneficial for malaria prevention and control (Asale et al., 2019; Von Seidlein et al., 2019). The success of such interventions is dependent on the level of education and awareness of malaria prevention tools among community members (Mutero et al., 2020). Studies conducted in Tanzania (Maheu-Giroux and Castro, 2013; Chaki et al., 2012), Rwanda (Hakizimana et al., 2022), Burkina Faso (Dambach et al., 2019), Bioko Island (García et al., 2022) and Malawi (Van den Berg et al., 2018; Phiri et al., 2021) have demonstrated the effectiveness of involving community members in routine surveillance, larviciding, and house improvements for vector control. Based on these studies, community engagement has the potential to play a significant role in successful implementation of vector control programs in other African countries.

In Tanzania, the focus of malaria vector control has been on ITNs and IRS (Kramer et al., 2017; Renggli et al., 2013; Mashauri et al., 2013; Kakilla et al., 2020). The 2014–2020 National Malaria Strategic Plan (Ministry of Health and Social Welfare, 2024) also recommended deploying larviciding in urban areas in line with the WHO guidelines (World Health Organization, 2019; World Health Organization, 2013), but this has recently been extended to rural areas (Mapua et al., 2021; MAELEZO TV, 2017; The United Republic of Tanzania Ministry of Health, C. D. G. E. and C, 2020). However, a recent study found limited community knowledge about larviciding and other challenges, including insufficient funding and technical expertise (Mapua et al., 2021), which may hinder the sustainability of the program in rural settings where malaria burden is higher. As in the urban settings, larviciding program in rural Tanzania involves the application of biolarvicides to all water bodies, which is a massive undertaking that severely constrains the program due to limited resources. The expansion of larviciding programs to rural communities in particular requires a deeper understanding of the ecology of the dominant malaria vector species, since the habitats may be more expansive and more numerous than as envisioned in the current WHO guidelines (World Health Organization, 2019; World Health Organization, 2013).

Previous entomological studies in rural south-eastern Tanzania, where *An. arabiensis* and *An. funestus* are the predominant malaria vectors, have shown that the latter species accounts for over 90 % of the ongoing malaria transmission (Kaindoa et al., 2017; Lwetoijera et al., 2014). *An. funestus* is also highly resistant to common public health pesticides targeting adult mosquitoes (Kaindoa et al., 2017; Pinda et al., 2020) thus requiring alternative control options (e.g. the use of the biocontrol agents such as, *Bacillus thuringiensis israelensis* (*Bti*) or *Lysinibacillus sphaericus* (*Ls*)). While preliminary results indicated that the aquatic habitats of immature *An. funestus* can be indeed few (i.e., only a small fraction of habitats are occupied by *An. funestus*), fixed (i.e., they tend to be permanent or semi-permanent) and findable (i.e., have unique characteristics allowing for ease of identification) (Nambunga et al., 2020). Expanded investigations (Msugupakulya et al., Unpublished data) suggest a more expansive range of habitats in different seasons, meaning that any effective control would require extensive engagement with locals to effectively search, characterize and target the habitats.

The aim of this study is to evaluate methods for involving community members in identifying, characterizing, and targeting the primary aquatic habitats of the dominant malaria vector species using biolarvicide, and to assess the impact of such an intervention on the density of the most relevant vector species in rural areas of Tanzania. This study focused on villages in the Kilombero valley, southeastern Tanzania, a meso-endemic community where *An. funestus* accounts for over 90 % of malaria transmission events.

## 2 Methods

### 2.1 Study area

**Fig. 1:**
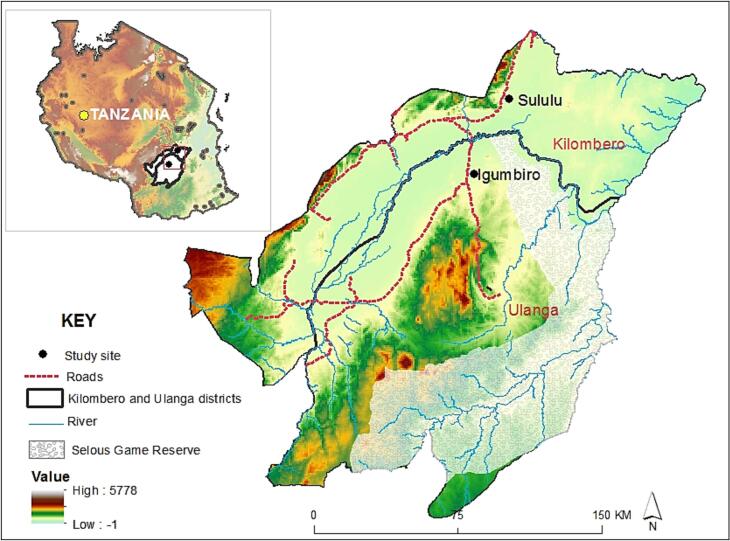
Map showing two villages in the Kilombero Valley, south-eastern Tanzania where the study was conducted.

The study was done in two villages, Igumbiro (8.35021°S, 36.67299°E) and Sululu (8.00324°S, 36.83118°E), both located on the Kilombero valley flood plain, south-eastern Tanzania (Fig. 1). Igumbiro is at a slightly higher altitude (340 m) above sea level than Sululu (315 m above sea level). Annual rainfall ranges from 1200 mm to 1400 mm, and the temperature ranges from 20 °C to 32 °C in these villages (Gebrekidan et al., 2020; Msofe et al., 2019). The majority of residents are subsistence rice farmers, but other crops such as sweet potatoes, beans, and maize are also produced (Gebrekidan et al., 2020).

### 2.2 Longitudinal entomological surveillance of adult mosquitoes

Mosquitoes were routinely collected in the two villages during the same period from March 2020 to September 2021. Three different households were sampled nightly in each village for four nights in a week, with one additional household sampled once per week as a sentinel station. The collections were done using CDC light traps from 6 pm to 6 am for host-seeking mosquitoes (Mboera et al., 1998) and Prokopack aspirators from 6 am to 7 am for resting mosquitoes (Maia et al., 2011). Collected female mosquitoes were sorted by taxa and physiological state (Gillies and De Meillon, 1968). Recent studies in these settings had determined that the *An. gambiae* s.l. and *An. funestus* group consisted of *An. arabiensis* (100 %) and *An. funestus* s.s. (>93 %) respectively (Kaindoa et al., 2017; Ngowo et al., 2017; Ngowo et al., 2021), but also that majority of the transmission (>90 %) is driven by *An. funestus* s.s. *(*Kaindoa et al., *2017**;* Mapua et al., *2022a**)*.

### 2.3 Recruitment and training of community volunteers

A total of 300 adult community members, 150 from each village, were randomly selected using residents' lists provided by village authorities. An interviewer-administered questionnaire, conducted in Swahili using KoboToolbox software (Harvard Humanitarian Initiative, 2024), assessed the community members' prior knowledge, awareness, and perception of disease-transmitting mosquitoes and larviciding. The questionnaires were administered following written informed consent. From these, ten community members from each village, meeting specific criteria, were selected for entomological training with an emphasis on equal gender distribution. The criteria were: i) ability to read and write properly, ii) involvement of the participant's household in the entomological surveillance, iii) residency in the village for at least two years, and iv) age between 18 and 50 years.

**Fig. 2:**
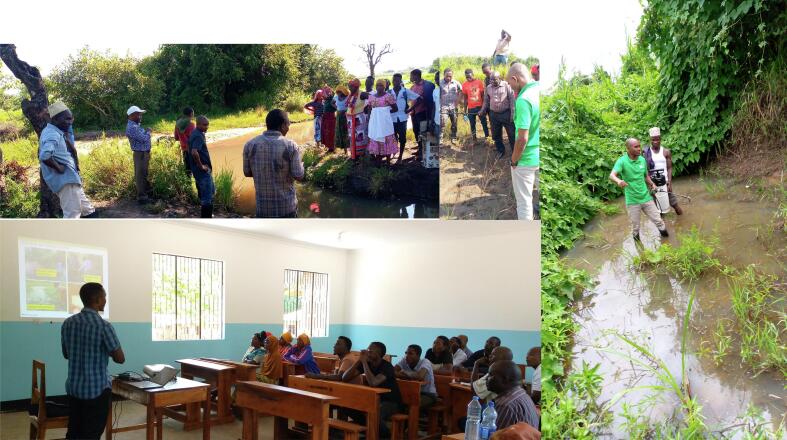
Theoretical and practical training of the community members for identification and characterization of aquatic habitats and application of larviciding.

**Fig. 3:**
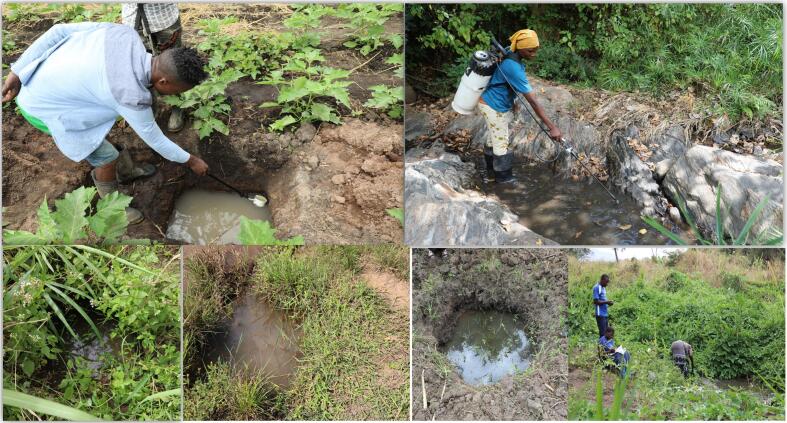
Example of the common *An. funestus* larvae habitats found in Sululu and Igumbiro villages, south-eastern Tanzania.

The training program for community volunteers was comprehensive, covering essential topics related to mosquito larvae identification, GPS usage, and the application of biolarvicides following WHO recommendations (World Health Organization, 2019; World Health Organization, 2013). It consisted of both theoretical and practical components. The theoretical aspect involved presentations and demonstrations (Fig. 2) on mosquito life stages, the biology of malaria vectors, and malaria transmission. The practical aspect included field visits, where volunteers engaged in hands-on training to practice identifying and characterizing mosquito larvae habitats, using GPS for mapping, and applying biolarvicides. Additionally, the practical sessions covered sampling techniques, estimating larval density, processing and storing immature mosquitoes, and recording data. (See Fig. 3.)

The training spanned three full days, with each day divided into theoretical sessions in the morning and practical sessions in the afternoon. Over the course of three days, three simplified modules were covered: i) an introduction to the control and biology of malaria vectors, ii) the identification and characterization of aquatic habitats of malaria vectors in Ulanga and Kilombero districts, and iii) larval source management. Facilitators from the Ifakara Health Institute and the government-owned biolarvicide plant (Tanzania Biotech LTD) led the training, ensuring high-quality instruction and relevance to local contexts. District malaria focal persons participated as liaisons, enhancing the connection between the community, government, and the Ifakara Health Institute. Although a formal post-assessment of the training program to evaluate the knowledge and skills acquired by community members was not conducted due to budget and timeline constraints, participants' skills were continuously assessed through hands-on activities during the practical sessions.

### 2.4 Identification and characterization of the aquatic habitats of the dominant malaria vectors, *Anopheles funestus*

The trained community members were deployed in their respective villages, with 2 km transects allocated to each participation for the identification and characterization of water bodies. Based on our previous survey (Nambunga et al., 2020), the focus was on types of habitats shown to be favored by *An. funestus* mosquitoes. For each aquatic habitat, a 350 ml larval dipper was used to check for the absence or presence of *An. funestus* larvae based on WHO larval survey guidelines (World Health Organization, 2013). Larvae counts and mosquito species identification were recorded per dipper sample in a square meter habitat. Each dipper was treated as an individual observation. Habitat types were defined, and environmental features were recorded. Physicochemical parameters (temperature (°C), total dissolved solid (ppm), acidity (pH), electrical conductivity (μS/cm) and dissolved oxygen (ppm)) were also measured, and GPS coordinates were recorded for each habitat.

### 2.5 Application and monitoring of the efficacy of *Bacillus thuringiensis israelensis* (*Bti*)

Following the habitat characterization and larval surveys, the trained community members applied *Bti* (i.e., 10 ml in liquid formulation for every square meter) to all aquatic habitats within the 2 km transect that has been identified as including *An. funestus* larvae. Our study employed *Bti* serotype H-14, a product registered with both the Central Pesticide Registration of Cuba and the Tropical Research Institute of Tanzania. This serotype is manufactured under license by the LABIOFAM Enterprise Group and sourced from Tanzania Biotech LTD, a local manufacturer. It is commercially available under the name BACTIVEC® (ITU 1200/mg). Application of the larvicide in both villages was done using the IK Vector Control Super Pressurized sprayers. This was followed by larval monitoring surveys after 24 h, 7, 23 and 30 days. Data from the larval surveys conducted during aquatic habitat characterization and after application were considered to enable pre and post intervention assessment.

### 2.6 Data analysis

The open-source software, R programming language version 3.6.0 (R Development Core Team, 2019) was used for the statistical analyses. Linear mixed effect models were used to assess the association between number of female adult mosquitoes collected per night per house, and status of the larviciding intervention (i.e., pre-larviciding and post-larviciding) in each village. In addition, households were used as a random effect. The linear mixed effect model was fitted using *lmer* function found within *lme4* package, whilst the variance estimator was set to Restricted Maximum Likelihood (REML) (Bates et al., 2014). Similar modelling was performed to determine association between number of larvae per habitat per village and status of larviciding, with the community member names as the random effect.

The questionnaire survey data was retrieved from KoboToolbox, cleaned, and coded for easier statistical analyses in R programming language software. The data was used to determine percentage of the community members that had knowledge and awareness of larviciding intervention, but also their perceptions towards larviciding. The Generalized Linear Model (GLM) was also used to investigate the association between community members perception towards larviciding intervention and their socio-demographic characteristics such as gender, age and literacy status.

GLMMs were used to determine the relationship between presence or absence of mosquito larvae and environmental characteristics. Highly correlated independent variables were removed, and the full model was fitted using a *glmer* function with binomial distribution and *logit* link function. Model selection was performed by removing insignificant terms. Final models were validated by assessing average residuals against expected values using binned residual plots. The same approach was applied to reveal the association between physicochemical attributes and occurrence of *An. funestus* larvae and other mosquitoes.

## 3 Results

### 3.1 Knowledge, awareness and perception of community members towards larviciding

A total of 300 community members (46 % women and 54 % men) participated in the knowledge assessment on larviciding. Of these, only 39 % were aware of larviciding as a malaria control strategy, a majority of these being men (Table 3). While age of participants did not influence their knowledge, those with secondary education were more than five times more knowledgeable than those without formal education (*p* < 0.001). Most of the participants had become aware of larviciding from family members or friends (Table 1). A large proportion (86 %) of the participants reported that they had never observed larviciding being implemented in their respective villages. While 8–20 % of the participants believed that larvicides were potentially harmful to other organisms (insects, large animals, and fish), more than 80 % agreed that larviciding could be necessary against disease-transmitting mosquitoes. Indeed, 71 % were willing to participate in the implementation of larviciding programs in their communities (Table 2).

**Table 1:** Knowledge and awareness of larviciding in the communities (*N* = 300).

Variable	Response	Percentage (n)
Awareness of larviciding (*N* = 300)	Yes	39 (117)
	No	61 (183)
Source of information (*N* = 117)	Family/Friends	65 (76)
	Radio/Television	14.5 (17)
	Village meeting	5.1 (6)
	Village health officer	6 (7)
	Village agricultural officer	0 (0)
	Malaria focal person	6 (7)
	Other sources	13.7 (16)
	Do not remember	1 (1)
Have you ever witnessed larviciding implemented in the community (N = 300)	Yes	8 (24)
	No	86 (258)
	Do not remember	6 (18)
Where are these larvicides manufactured (*N* = 300)	Domestic	6.7 (20)
	Abroad	6.7 (20)
	Both	1.3 (4)
	Do not know	85.3 (256)
Which is the first stage during larvicides application (N = 300)	Identification of aquatic habitats	29 (87)
	Community sensitization	28.3 (85)
	Estimation of larvicide quantity	1.7 (5)
	Spraying larvicides	3 (9)
	Other	3.3 (10)
	Do not know	34.7 (104)

**Table 2:** Perception of community members towards larviciding for the malaria prevention (*N* = 300).

Variable	Response	Percentage (n)
Have you ever participated in larviciding (*N* = 300)	Yes	1.3 (4)
	No	98.7 (296)
Do you think larvicides are harmful to insect (*N* = 300)	Yes	20 (60)
	No	31.3 (94)
	Do not know	48.7 (146)
Do you think larvicides are harmful to fish (N = 300)	Yes	8 (24)
	No	39.3 (118)
	Do not know	52.7 (158)
Do you think larvicides are harmful to animal (N = 300)	Yes	12.3 (37)
	No	40.7 (122)
	Do not know	47 (141)
Willingness to participate in larviciding (N = 300)	Yes	71 (213)
	No	29 (87)
Acceptance of larviciding (N = 300)	Agree	82.3 (247)
	Do not agree	4.7 (14)
	Neutral	13 (39)

**Table 3:** Association between socio-demographic characteristics and community perception towards larviciding.

Category	Variable	Odds ratio (95 % CI)	*P*-value
Sex	Female	1	–
	Male	1.84 (1.13–2.99)	0.01
Age category (in years)	18–25	1	–
	26–35	0.87 (0.46–1.68)	0.69
	36–45	0.66 (0.32–1.37)	0.26
	46–50	0.50 (0.15–1.59)	0.24
	Above 50	0.63 (0.32–1.21)	0.17
Education level	No formal education	1	–
	Primary	1.51 (0.85–2.68)	0.16
	Secondary	5.40 (2.20–13.24)	<0.001

**Table 4:** Characteristics of aquatic habitats of *Anopheles funestus* and other mosquito species.

Parameter		No. habitats observed (%)	Habitats without larvae	Habitats *with An. funestus* larvae (%)	Habitats with other *Anopheles* larvae n(%)	Habitats with Culicine sp. larvae n(%)
Water movement	Stagnant	370 (49.7)	7 (1.9)	167 (45.1)	110 (29.7)	123 (33.2)
	Slow	354 (47.6)	11 (3.1)	164 (46.3)	90 (25.4)	147 (41.5)
	Fast	20 (2.7)	1 (5)	11 (55)	1 (5)	7 (35)
Water type	Permanent	525 (70)	10 (1.9)	279 (53.1)	150 (28.6)	230 (43.8)
	Temporary	219 (29.4)	9 (4.1)	63 (28.8)	51 (23.3)	47 (21.5)
Water colour	Clear	520 (69.9)	16 (3.1)	224 (43.1)	136 (26.2)	196 (37.7)
	Coloured	161 (21.6)	3 (1.8)	89 (55.3)	46 (28.6)	57 (35.4)
	Polluted	63 (8.5)	0	29 (46)	19 (30.2)	24 (38.1)
Habitat type	Swamp	168 (24.6)	2 (1.2)	97 (57.7)	41 (24.4)	76 (45.2)
	Stream	390 (56.2)	12 (3.1)	185 (47.4)	91 (23.3)	157 (40.3)
	Rice field	10 (1.6)	0	4 (40)	4 (40)	7 (70)
	Natural well	43 (3.7)	5 (11.6)	16 (37.2)	10 (23.3)	17 (39.5)
	Man-made well	32 (2.3)	0	7 (21.8)	7 (21.8)	3 (9.4)
	Puddle	101 (11.6)	0	33 (32.7)	48 (47.5)	17 (16.8)
Shade over habitat	None	425 (56.2)	10 (2.4)	196 (46.1)	127 (29.9)	145 (34.1)
	Partial	236 (31.9)	9 (3.8)	102 (43.2)	55 (23.3)	95 (40.3)
	Heavy	83 (11.8)	0	44 (53)	19 (22.9)	37 (44.6)
Vegetation quantity	None	257 (34.5)	12 (4.7)	114 (44.4)	57 (22.2)	96 (37.4)
	Scarce	68 (9.1)	0	34 (50)	15 (22)	27 (39.7)
	Moderate	217 (29.2)	6 (2.7)	103 (47.5)	57 (26.3)	78 (35.9)
	Abundant	202 (27.2)	1 (0.5)	91 (45.1)	72 (35.6)	76 (37.6)
Vegetation type	None	121 (16.3)	0	74 (61.2)	33 (27.3)	69 (57)
	Submerged	46 (6.2)	0	23 (50)	25 (54.3)	8 (17.4)
	Floating	99 (13.3)	0	70 (70.7)	34 (34.3)	53 (53.5)
	Emergent	478 (64.2)	19 (4)	175 (36.6)	109 (22.8)	147 (30.8)
Algae quantity	None	516 (69.4)	17 (3.3)	197 (26.5)	126 (16.9)	171 (23)
	Scarce	48 (6.5)	0	34 (70.8)	20 (41.7)	17 (35.4)
	Moderate	129 (17.3)	0	85 (65.9)	40 (31)	66 (51.2)
	Abundant	51 (6.9)	2 (3.9)	26 (51)	15 (29.4)	23 (45.1)
Habitat depth	Less than 10 cm	361 (48.5)	15 (4.2)	196 (54.3)	72 (19.9)	178 (49.3)
	Between 10 and 50 cm	355 (47.7)	4 (1.1)	138 (38.9)	117 (33)	96 (27)
	Greater than 50 cm	28 (3.8)	0	8 (28.6)	12 (42.9)	3 (10.7)

No. habitats observed represents total number of observations per parameter.

Rice field represents habitats specifically found within non-irrigational rice fields.

**Table 5:** Results of multivariate regression analysis of habitat characteristics and mosquito larvae.

Parameter		*Anopheles arabiensis*		*Anopheles funestus*		*Culex* spp.	
		OR (95 % CIs)	*p*-value	OR (95 % CIs)	*p*-value	OR (95 % CIs)	*p*-value
Water movement	Stagnant	1	–	1	–	1	–
	Slow	2.21 (0.60–8.17)	0.23	37.83 (6.99–204.81)	<0.001	0.59 (0.07–5.31)	0.64
	Fast	0.77 (0.12–4.99)	0.79	21.25 (2.82–160.02)	<0.05	0.61 (0.05–6.89)	0.69
Water type	Temporary	1	–	1	–	1	–
	Permanent	0.14 (0.01–1.87)	0.14	1.04 (0.14–7.91)	0.97	0.21 (0.01–3.73)	0.29
Water colour	Clear	1	–	1	–	1	–
	Coloured	1.44 (0.72–2.91)	0.30	1.97 (0.97–4.01)	0.06	0.88 (0.44–1.76)	0.72
	Polluted	0.71 (0.29–1.76)	0.46	1.78 (0.73–4.32)	0.20	1.20 (0.51–2.84)	0.68
Habitat type	Swamp	1	–	1	–	1	–
	Stream	0.35 (0.14–0.83)	0.02	6.89 (3.33–14.26)	<0.001	0.10 (0.04–0.27)	<0.001
	Rice field	1.17 (0.22–6.32)	0.85	9.49 (1.71–52.52)	<0.05	0.08 (0.01–0.76)	0.03
	Natural well	0.10 (0.03–0.36)	<0.001	4.26 (1.47–12.38)	<0.05	0.10 (0.03–0.38)	<0.001
	Man-made well	0.40 (0.11–1.44)	0.16	9.66 (3–31.04)	<0.001	0.09 (0.02–0.37)	<0.001
	Puddle	0.32 (0.11–0.91)	0.03	10.26 (4.02–26.16)	<0.001	0.07 (0.02–0.23)	<0.001
Shade over habitat	None	1	–	1	–	1	–
	Partial	0.62 (0.19–2.02)	0.43	1.72 (0.54–5.44)	0.36	1.01 (0.37–2.74)	0.98
	Heavy	0.48 (0.13–1.75)	0.27	1.78 (0.52–6.16)	0.36	0.57 (0.19–1.73)	0.32
Vegetation type	None	1	–	1	–	1	–
	Floating	2.65 (1.31–5.38)	<0.05	0.52 (0.24–1.11)	0.09	0.44 (0.21–0.89)	0.02
	Submerged	2.09 (0.95–4.61)	0.07	1.19 (0.53–2.69)	0.68	0.28 (0.11–0.75)	0.01
	Emergent	0.07 (0–1.09)	0.06	0.17 (0.02–1.50)	0.11	0.03 (0–0.68)	0.03
Vegetation quantity	None	1	–	1	–	1	–
	Scarce	0.27 (0.02–3.70)	0.33	0.35 (0.04–3)	0.34	0.08 (0–1.49)	0.09
	Moderate	0.17 (0.01–2.14)	0.17	0.49 (0.61–3.96)	0.50	0.11 (0–1.92)	0.13
	Abundant	0.14 (0.01–1.84)	0.14	0.31 (0.04–2.47)	0.27	0.07 (0–1.16)	0.06
Algae quantity	None	1	–	1	–	1	–
	Scarce	0.29 (0.06–1.32)	0.11	1.85 (0.56–6.10)	0.32	0.15 (0.04–0.60)	<0.05
	Moderate	0.76 (0.21–2.71)	0.67	2.32 (0.79–6.83)	0.13	0.18 (0.05–0.63)	<0.05
	Abundant	0.82 (0.21–3.23)	0.78	21.81 (0.56–5.89)	0.32	0.39 (0.10–1.497)	0.17
Habitat depth	Less than 10 cm	1	–	1	–	1	–
	Between 10 and 50 cm	2.87 (0.69–11.92)	0.15	116.34 (18.22–743.05)	<0.001	0.24 (0.03–2.24)	0.21
	Greater than 50 cm	1.02 (0.16–6.57)	0.98	138.11 (18.48–1031.95)	<0.001	0.12 (0.01–1.45)	0.10

**Table 6:** Results of multivariate regression analysis of different physicochemical characteristics and their association with occurrence of mosquito larvae.

Physicochemical characteristic	*Anopheles arabiensis*		*Anopheles funestus*		*Culex* spp.	
	OR (95 % CIs)	*p*-value	OR (95 % CIs)	*p*-value	OR (95 % CIs)	*p*-value
pH	0.89 (0.32–2.43)	0.25	10.01 (1.11–90.32)	0.04	0.22 (0.06–0.83)	<0.001
Temperature (°C)	0.80 (0.62–1.02)	0.08	0.74 (0.35–1.54)	0.42	3.08 (1.82–5.23)	0.03
Electrical conductivity (μS/cm)	1.02 (1–1.04)	0.02	0.98 (0.94–1.02)	0.24	1.13 (1.04–1.23)	<0.001
TDS (ppm)	1 (0.98–1.02)	0.94	1.01 (0.96–1.06)	0.67	0.80 (0.66–0.97)	<0.05
DO (ppm)	0.96 (0.86–1.08)	0.53	0.83 (0.41–1.68)	0.60	1 (0.75–1.34)	1

### 3.2 Habitat characteristics and mosquito species in different aquatic habitats

A total of 360 aquatic habitats were surveyed, including 167 (46.4 %) in Sululu and 193 (53.6 %) in Igumbiro village (Table 4). Larvae of the dominant malaria vector, *An. funestus*, exhibited a preference for naturally occurring or man-made wells that receive spring-fed water, river streams, and puddles, rather than swamps (Table 5). *Culex* spp. and *An. arabiensis* larvae displayed comparable preferences, except for the latter, which did not favor man-made wells. Furthermore, *An. funestus* larvae were more commonly found in water bodies with slow (*p* < 0.001) flow rate compared to other mosquito species (Table 5).

The presence of *An. funestus* larvae was found to be independent of vegetation type or quantity. However, *An. arabiensis* showed a preference for habitats with floating vegetation (Table 5). Furthermore, habitats with vegetation were less likely to host *Culex* spp. larvae. Moreover, according to the modelling results, there was a positive correlation between the depth of aquatic habitats and the likelihood of encountering *An. funestus* larvae. *Culex* spp. larvae were negatively associated with the habitats having scarce or moderate algae quantity, but the same was not observed for other mosquito species (Table 5). The occurrence of mosquitoes was unaffected by the shading over the habitat, water clarity (i.e., clear, coloured, or polluted), or habitat permanency (Table 5).

Acidity (pH), temperature (°C), electrical conductivity (μS/cm), total dissolved solids (ppm) and dissolved oxygen (ppm) were measured in a total of 178 aquatic habitats in Igumbiro village. Temperature, pH, total dissolved solids, and electrical conductivity were found to have an impact on the occurrence of *Culex* spp. larvae. In contrast, only electrical conductivity affected the presence of *An. arabiensis*, while pH specifically influenced the presence of *An. funestus* (Table 6).

### 3.3 Effects of larviciding on the abundance of adult female mosquitoes and larvae

**Fig. 4:**
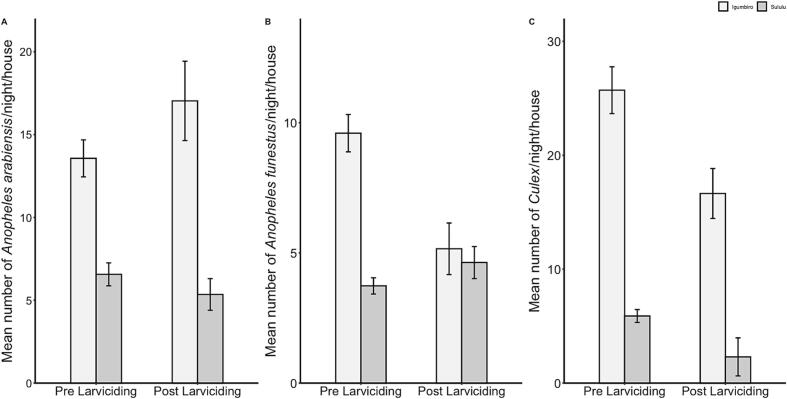
Effect of a single application of biolarvicides on abundance of adult *An. funestus*, *An. arabiensis* and *Culex* spp. inside houses in Sululu and Igumbiro villages, south-eastern Tanzania.

In Igumbiro, there was a significant reduction of indoor densities for *An. funestus* (*p* < 0.001) and *Culex* spp. (p < 0.001) following larviciding. However, the densities of *An. arabiensis* significantly increased after larviciding (*p* = 0.005). In Sululu, no significant change was observed in the densities of *An. arabiensis* (*p* = 0.268) or *An. funestus* (*p* = 0.119), but there was a significant decline in densities of *Culex* spp. (*p* = 0.035) (Fig. 4).

**Fig. 5:**
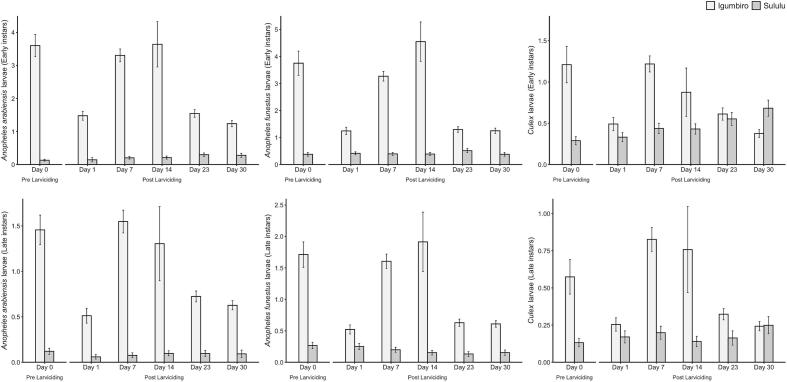
Densities of early- and late-instar larvae of *An. funestus*, *An. arabiensis* and *Culex* spp. before and after a single application of biolarvicides in Sululu and Igumbiro villages, south-eastern Tanzania. A previous study (Matowo et al., 2019) identified that *Culex* spp. mainly consisted of *Cx. pipiens pipiens* and *Cx. quinquefasciatus*.

For early instars larvae densities (Fig. 5), there was a significant reduction in the Igumbiro village for *An. arabiensis* (*p* < 0.001), *An. funestus* (p < 0.001) and *Culex* spp. (p < 0.001) but the densities remained statistically the same for *An. arabiensis* (*p* = 0.404) and *An. funestus* larvae (*p* = 0.651) in the Sululu village. The densities of *Culex* spp. at early instars were significantly increased after larviciding intervention (*p* = 0.001) in Sululu village (Fig. 5). At late instars, the densities of *An. arabiensis* (*p* < 0.001), *An. funestus* (p < 0.001) and *Culex* spp. (p < 0.001) were significantly reduced in Igumbiro village but not in Sululu, where only the *An. funestus* densities were marginally reduced (*p* = 0.045) after larviciding (Fig. 5).

## 4 Discussion

Effective engagement of community members can ensure sustainability of public health programmes (Holder et al., 2000; Das et al., 2014), in part by creating a sense of ownership and responsibility (Ingabire et al., 2017; Gubler and Clark, 1996). A recent study (Mapua et al., 2021) emphasized the importance of such engagement to improve the larviciding programs in rural Tanzania, following recent expansion of the practice beyond urban settings (The United Republic of Tanzania Ministry of Health, C. D. G. E. and C, 2020). This can be achieved through a combination of community sensitization meetings and targeted training of community-based persons, as demonstrated in urban Dar es Salaam, where community-owned resource persons previously enabled scale-up of larval source management (Maheu-Giroux and Castro, 2013; Geissbühler et al., 2009).

In rural Africa, where mosquito habitats can be more diverse and numerous, and where the existing infrastructure may be inadequate for traditional LSM approaches, a detailed understanding of the ecology of dominant malaria vectors is also necessary to optimize resource use. For example, in the Kilombero valley, Tanzania, where just one of the many vector species, *An. funestus*, now accounts for nine in every ten malaria infections (Kaindoa et al., 2017; Mapua et al., 2022b), it may be most appropriate to preferentially target habitats for this vector species. Previous studies in the area have provided a baseline for such investigations by mapping the primary characteristics of the vector species habitats (Nambunga et al., 2020) and also by highlighting the importance of such knowledge for practical LSM practices (Mapua et al., 2021). This study tested the potential of training community members to identify, characterize and target larval habitats of dominant malaria vectors in rural south-eastern Tanzania. Biolarvicides were applied to aquatic habitats of *An. funestus* mosquitoes in the two villages, and the immediate impact assessed by estimating the larval densities and also adult densities of the mosquitoes in people's dwellings.

The current larviciding program in mainland Tanzania is implemented by community health workers (i.e., secondary school graduates with a year of community health training) under the supervision of ward health officers. Limited biolarvicide supply and inadequate funding put severe constraints on implementation of the program, however. The present study has demonstrated the effectiveness of involving lay members of the community in identifying and characterizing aquatic habitats of the most competent malaria vector species and applying the same biolarvicides as provided by the government. Involving community members in larviciding is not uncommon in Tanzania (Maheu-Giroux and Castro, 2013; Geissbühler et al., 2009), but this study demonstrates the role they can play in a species-focused larviciding approach. The training of community members was effective, and they identified habitats and environmental characteristics associated with the occurrence of *An. funestus* larvae. The aquatic habitats identified by the trained community members had similar characteristics as those previously identified by expert vector biologists (Nambunga et al., 2020). The number of *An. funestus* mosquitoes inside the houses in the villages was significantly reduced after application of biolarvicides to the aquatic habitats identified by community members. The study suggests that relying on the community to sustainably implement the government-led larviciding program is possible, but recurring training would be better to maximize the program's impact. Additionally, Table 1, Table 2 present the initial awareness and perception of community members, which served as a basis for developing the training modules. Although the subset of community members that underwent training exhibited improved awareness and perception during and after the intervention, we did not seek to quantify this improvement due to the project's tight timeline. The ability of trained community members to carry out habitat identification and larviciding activities indicates that capacity for larval control can be enhanced through a set of simplified yet engaging training sessions.

However, it is important to note that the findings from this study may not be directly generalizable to other rural settings or different ecological zones. The study was conducted in two specific villages within the Kilombero Valley, which has unique environmental and ecological characteristics. Factors such as mosquito species composition, habitat types, and local community practices can vary significantly in different regions. Therefore, while the training program and larviciding approach demonstrated effectiveness in this context, further studies are necessary to evaluate the applicability and efficacy of similar interventions in other rural areas with different ecological conditions.

In the two villages investigated, more than 300 aquatic habitats were surveyed, and it was found that *An. funestus* larvae had a preference for habitats with slow or fast-moving waters, such as streams. While this has been previously been linked to the higher levels of dissolved oxygen and aeration in such waters (Nambunga et al., 2020; Tchigossou et al., 2017), this current study did not find any significant associations between dissolved oxygen levels and the presence of *An. funestus*. Similarly, while previous studies have reported the preference of *An. funestus* for vegetated habitats (Gillies and De Meillon, 1968; Gimnig et al., 2001; Cohuet et al., 2003), no such association was observed here. Except for pH, the physicochemical characteristics of aquatic habitats did not appear to have a significant association with the occurrence of *An. funestus* larvae, which was also observed in other mosquito species, particularly *Culex* spp. larvae. These differences may, in part, be due to the limited geographical extent of the current study, which covered only two villages. It was also observed that the main aquatic habitats of *An. funestus*, including streams and wells, were in close proximity to agricultural activities, suggesting they may be constantly exposed to agricultural pesticide wastes as previously observed in the area (Matowo et al., 2020), potentially exacerbating the challenge of insecticide resistance. Fortunately, the current larviciding programs in Tanzania deploy biolarvicides (i.e., *Bacillus thuringiensis israelensis* and *Lysinibacillus sphaericus*), which remain effective against pyrethroid-resistant malaria vectors.

While the approach tested here was clearly effective, we did not investigate the optimal timing of the larviciding. Instead, the single application was done in the dry season, when the habitats were least numerous and least expansive. Also, a recent mathematical simulation (Runge et al., 2021) suggested that the most effective timing for larviciding is during or at the beginning of the rainy season but that was on assumption that the main vector species would be *An. gambiae* s.s., which breed in temporary pools and whose populations peak during the wet season. It however remains unclear what the optimal timing for larviciding would be in areas dominated by *An. funestus*, which tends to occupy perennial habitats and therefore remains important throughout the year. The results of this study may be useful for future modelling exercises to assess such scenarios. Moreover, a distinct trend of aquatic habitat recolonization was observed within one to two weeks after treatment, followed by a notable reduction. This pattern suggests the residual effectiveness of *Bti*. Furthermore, it's important to keep in mind that our results are based on a single larvicide application, which served primarily to showcase the capability of trained community members in targeting disease-transmitting mosquitoes. This also highlights the success of a simpler yet effective training approach.

Tanzania has had great examples of cross-sectoral engagement for malaria prevention. In addition to the supply of locally manufactured biolarvicides from the Tanzania biotechnology industry, there have also been major investments in community sensitization and engagement. For example, the Dar es Salaam Urban Malaria Control Programme (UMCP) organized a highly effective community-based program of environmental management to reduce densities of malaria vectors, which even included a degree of fundraising by the communities (De Castro et al., 2004). More recently, the use of trained community owned resource persons to deploy the larvicides and also monitor adult densities resulted in significant declines of malaria in the city (Maheu-Giroux and Castro, 2013; Chaki et al., 2012; Geissbühler et al., 2009). This current study demonstrate that such community-based strategies can be expanded to rural settings such as the Kilombero Valley. This could significantly reduce implementation costs, especially as community members are generally willing to participate. We did not explicitly investigate the community's willingness to participate without compensation in the current study. Nevertheless, previous studies have indicated that such willingness is possible when there is a higher perceived level of safety and acceptance of the product (Hakizimana et al., 2022; Dambach et al., 2018). Already, the district-level malaria officials have been conducting community sensitization programs to support larval source management (Mapua et al., 2021). However, it is evident that more efforts are needed given that significant proportions of the rural community members (∼60 % in this study), remain unaware of the importance of larviciding for malaria control.

Another important observation was that while *An. funestus* clearly prefer certain habitat types, they do often cohabit with other mosquito species. In this study the application of the biolarvicides in the habitats of *An. funestus* also reduced indoor densities of adult *Culex* spp. but not *An. arabiensis*. Also, though *An. arabiensis* preferred irrigational rice fields, their larva densities were also reduced in habitats that they shared with *An. funestus*. It can be assumed therefore that the current strategy of applying larvicides to all aquatic habitats could be effective as well, especially if there is adequate resources and manpower. However, the findings also indicate that it might be more cost-effective to preferentially target the most competent malaria vector species.

This study also had some limitations. Firstly, the use of a 2 km transects underestimated the potential effectiveness of larviciding because mosquitoes can fly much longer, sometime up to or beyond 4 km (Gillies, 1961). This means that mosquitoes could have emerged from habitats beyond the transect, potentially reducing the larviciding efficacy. Additionally, the study did not gather post-assessment feedback from community members who participated in the program, primarily due to constraints related to the project timeline and available resources. Their views and insights could have provided valuable information on the sustainability of the approach and identified areas for improvement. Furthermore, the study focused solely on assessing the impact of larviciding on the abundance of the major malaria vector, without evaluating its effect on malaria prevalence in the villages. A recent simulation study (Runge et al., 2021) suggests a reduction in malaria prevalence one month after larvicide application. However, due to time limitations, the larvicides were only applied once in this study. While this may have been sufficient for demonstrating the role of community members, it is itself inadequate for assessing efficacy of the intervention on its own. Lastly, the application of biolarvicides was limited to larval habitats that tested positive for *An. funestus* during the survey, potentially reducing coverage by not treating habitats that could have harbored *An. funestus* larvae but tested negative at the time of the survey.

In addition to these limitations, potential biases must be considered. Selection bias may have been introduced during the recruitment of volunteers for the entomological training. The criteria for selecting participants—such as the ability to read and write properly, involvement in household entomological surveillance, a minimum residency of two years, and an age range of 18 to 50 years may have excluded certain community members who could have contributed valuable perspectives. This selection process might have favored more educated or engaged individuals, potentially skewing the results. Additionally, response bias in the questionnaires cannot be ruled out. Since the questionnaires were administered by interviewers, there is a possibility that respondents provided socially desirable answers rather than their true perceptions and knowledge levels about disease-transmitting mosquitoes and larviciding. This could lead to an overestimation of the community's baseline awareness and perception. To mitigate these biases in future studies, a more inclusive selection process and the use of anonymous self-administered questionnaires could be considered.

## 5 Conclusion

Previous studies have shown the effectiveness of larviciding in urban areas of Tanzania, following WHO guidelines. However, the National Malaria Strategic Plan of Tanzania has extended larviciding to rural areas, despite not strictly adhering to these guidelines. This study highlights the potential of species-focused community-led larviciding as a sustainable intervention for malaria control in rural settings. The observed reduction in mosquito densities demonstrates that, with proper training and community engagement, local communities can successfully implement and maintain vector control strategies. These findings suggest that this approach can be effectively adapted to other resource-limited rural settings. Furthermore, by reducing reliance on centralized programs, this model promotes self-sufficiency and community ownership, which are critical for the long-term sustainability of malaria control efforts. The results have important implications for policy makers and public health officials, as community-led interventions could complement existing vector control strategies, thereby enhancing the overall impact of malaria control programs.

The following are the supplementary data related to this article.

Binned residual plot for Anopheles funestus model Supplementary Fig. S1

Binned residual plot for Culex spp. model Supplementary Fig. S2

Binned residual plot for Anopheles arabiensis model Supplementary Fig. S3

## Ethics approval and consent to participate

Ethical approvals for this project were obtained from Ifakara Health Institute's Institutional Review Board (Protocol ID: IHI/IRB/No: 29–2019) and the Medical Research Coordinating Committee (MRCC) at the National Institute for Medical Research, in Tanzania (Protocol ID: NIMR/HQ/R.8a/Vol.IX/3517). Written consents were sought from all participants of this study, after they had understood the purpose and procedure of the discussions.

## Consent for publication

Permission to publish this study was obtained from National Institute for Medical Research, in Tanzania (No: NIMR/HQ/P. 12 VOL.XXXVI 37).

## Funding

This study was supported by the 10.13039/100010269Wellcome Trust International Masters Fellowship in Tropical Medicine and Hygiene (Grant No. 212633/Z/18/Z) awarded to SAM, Bill and Melinda Gates Foundation (Grant Number: OPP1177156) awarded to FOO. All grants were held at Ifakara Health Institute.

## Author contributions

SAM, JL, FT and FOO were involved in study design. SAM, AJL, IHN and KK were involved in data collection. SAM conducted data analysis. DK contributed in designing and validating the model selection part of the data analysis. SAM and AJL wrote the manuscript. KU, SM and GJ facilitated training of the community members and vector surveillance officers, and collaboration with district's malaria focal persons. FOO, JL, FT, DK, KU, GJ, WM, IHN and SM provided thorough review of the manuscript. All authors read and approved the final manuscript.

## CRediT authorship contribution statement

**Salum A. Mapua:** Writing – review & editing, Writing – original draft, Project administration, Methodology, Investigation, Funding acquisition, Formal analysis, Data curation, Conceptualization. **Alex J. Limwagu:** Writing – original draft, Project administration, Methodology, Investigation, Data curation. **Dmitry Kishkinev:** Validation, Formal analysis. **Khamis Kifungo:** Project administration, Investigation. **Ismail H. Nambunga:** Methodology, Investigation. **Samuel Mziray:** Project administration, Methodology. **Gwakisa John:** Project administration, Methodology. **Wahida Mtiro:** Project administration, Methodology. **Kusirye Ukio:** Project administration, Methodology. **Javier Lezaun:** Writing – review & editing, Writing – original draft, Visualization, Validation, Supervision, Methodology, Investigation, Funding acquisition, Formal analysis, Data curation, Conceptualization. **Frederic Tripet:** Writing – review & editing, Writing – original draft, Visualization, Validation, Supervision, Methodology, Investigation, Funding acquisition, Formal analysis, Data curation, Conceptualization. **Fredros O. Okumu:** Writing – review & editing, Writing – original draft, Visualization, Validation, Supervision, Resources, Methodology, Investigation, Funding acquisition, Formal analysis, Data curation, Conceptualization.

## Declaration of competing interest

The authors declare no competing interests.

## Data Availability

The data will be made available by the corresponding author upon reasonable request.
