# Supplementary figures and images for: Empowering rural communities for effective larval source management: A small-scale field evaluation of a community-led larviciding approach to control malaria in south-eastern Tanzania
Source: Parasite Epidemiol Control. 2024 Oct 4;27:e00382. doi: 10.1016/j.parepi.2024.e00382 (PMC11493201; doi:10.1016/j.parepi.2024.e00382)

Binned residual plot

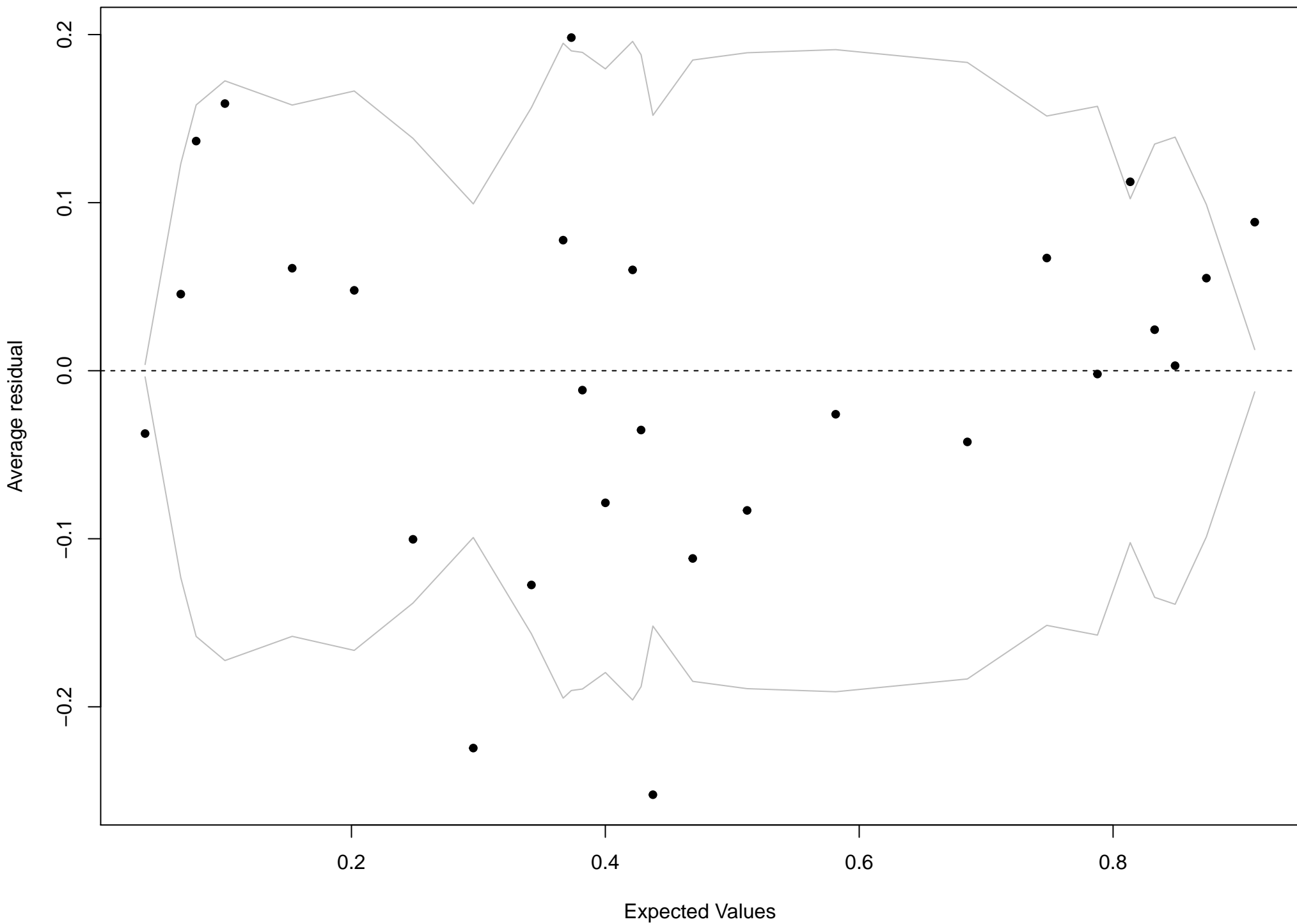

Supplement: Binned residual plot for Anopheles funestus model [file mmc1.pdf]

Binned residual plot

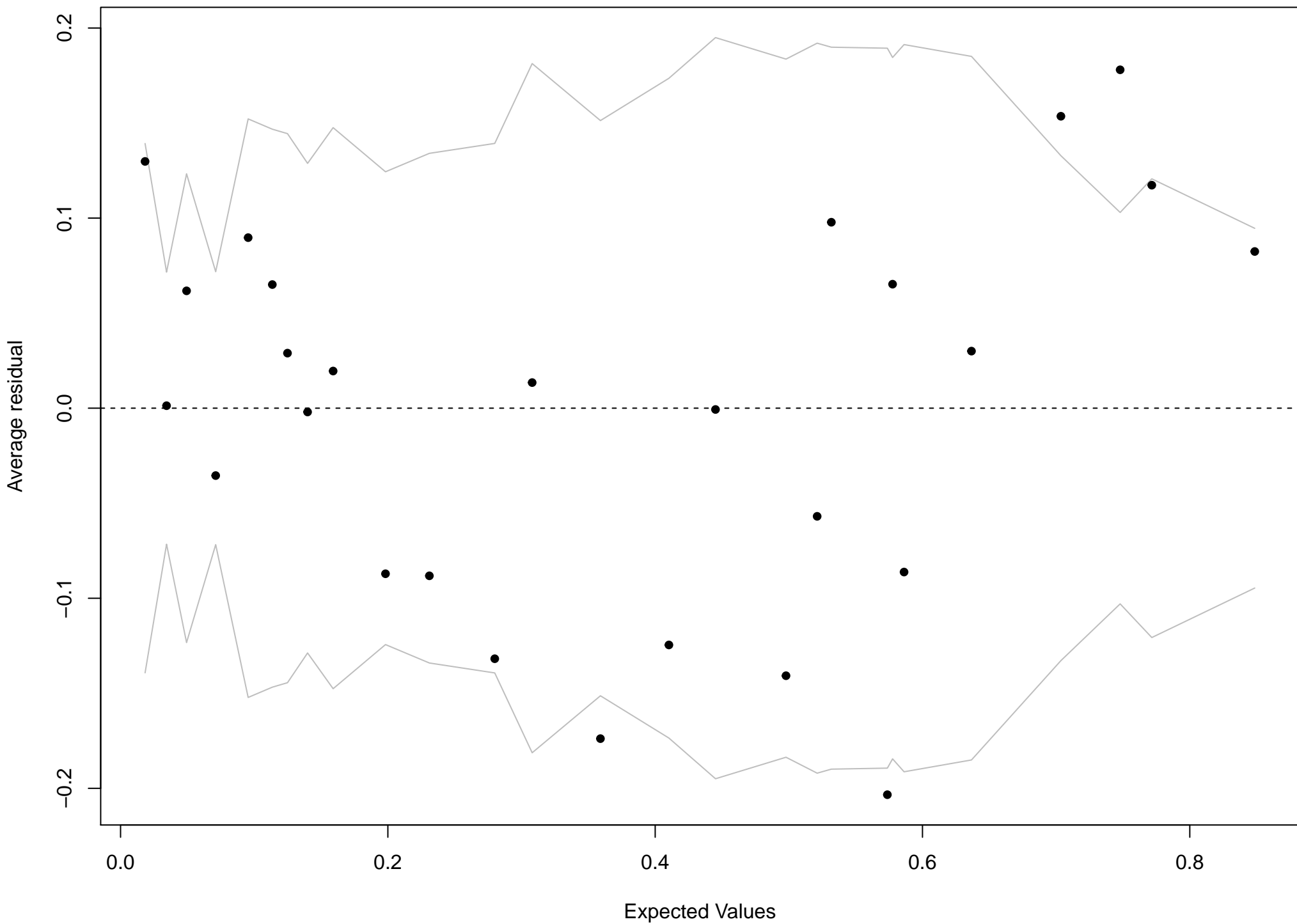

Supplement: Binned residual plot for Culex spp. model [file mmc2.pdf]

Binned residual plot

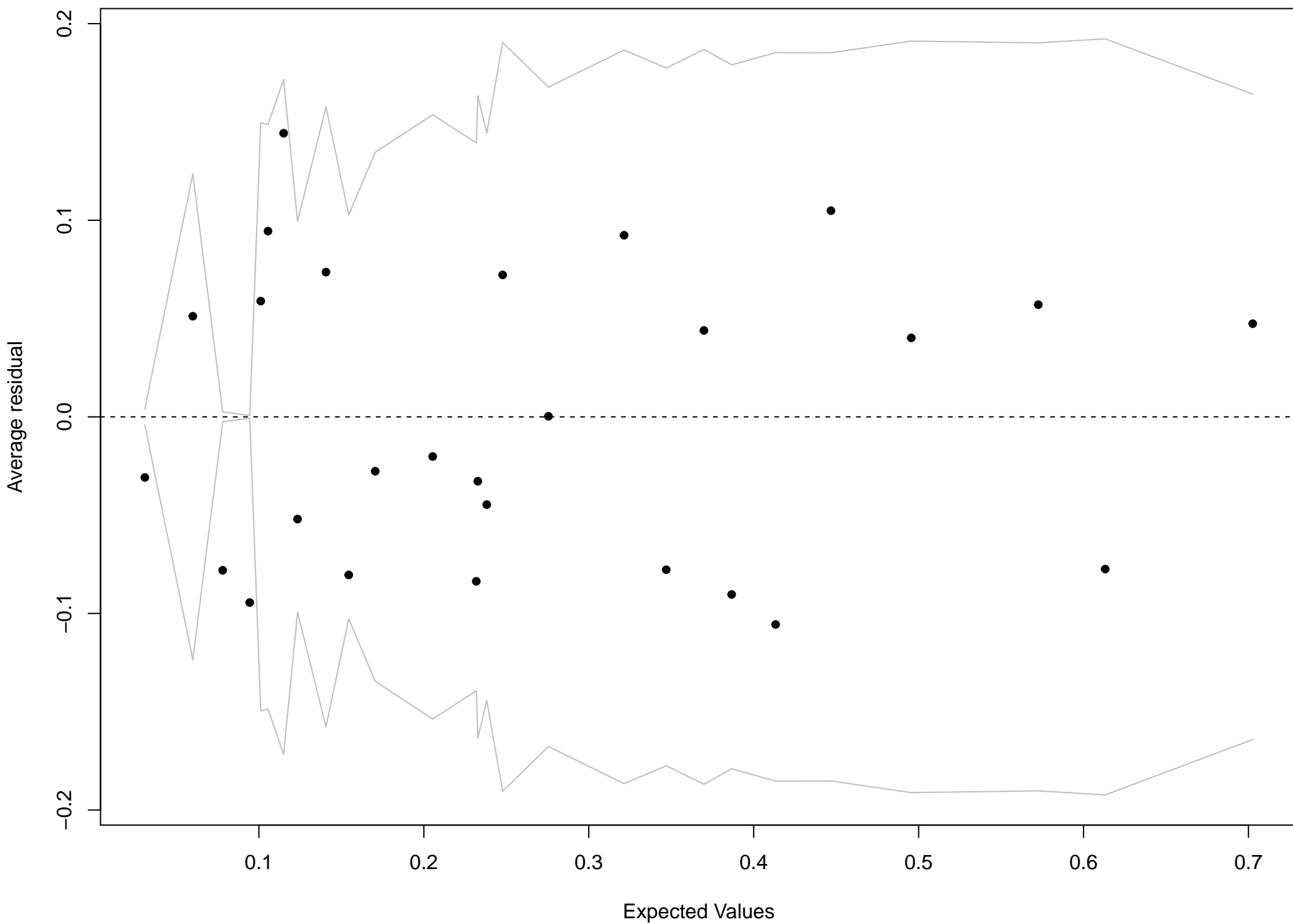

Supplement: Binned residual plot for Anopheles arabiensis model [file mmc3.pdf]
